# Telehealth and primary care: a special collection from BJGP Open

**DOI:** 10.3399/BJGPO.2022.0120

**Published:** 2022-10-05

**Authors:** Sarah J White, Hajira Dambha-Miller

**Affiliations:** 1 Centre for Social Impact, UNSW, Sydney, Australia; 2 BJGP Open, Royal College of General Practitioners, London, UK

**Keywords:** Telehealth, Primary care, General practice, Telemedicine, Remote consultation

In this *BJGP Open* special issue, we explore how increased reliance on telehealth has changed clinical practice. Telehealth, defined as the provision of healthcare remotely through telecommunications technology, has been integrated into health services with varying degrees of success in the past. The COVID-19 pandemic has accelerated this process, with telehealth presenting a solution to care delivery during national lockdowns and social distancing requirements. There has been vast amounts of research examining what has worked, potential improvements, and what should be retained in the post-pandemic world. This collection of articles adds to the discourse and considers telehealth in three broad areas: access to services, quality of care, and conducting consultations.

A central suggestion in favour of continued investment in telehealth is the increased access that it provides to services. Three of the articles in this collection report on improvements in access through telehealth, while also acknowledging that increased planning and investment at a system level is required to ensure usability and equitability.^1–3^ Some of these barriers to access are not unique to telehealth; consideration of the additional complexity that telehealth can present to accessing care is essential. When the system is designed to support telehealth access, the benefits for patients are realised, as shown by Bhatti *et al*.^4^

This leads on to our articles on the quality of care when using telehealth. Variations in care quality are a continued concern for what has become an expanded and extended use of telehealth services. Some care is not substantively impacted by its use,^5^ however continued examination of differences in practice are needed to support safe, high quality telehealth care.^6^ Rosen *et al* and Shaw *et al* explore ways of mitigating risk for effective use of telehealth, particularly through inclusion and support strategies.^7,8^ Similar to increased access, these strategies require system level support to adequately maintain quality of care across different modes of delivery.

At a more local level, conducting consultations in a way that provides quality of care is important for the ongoing integration of telehealth into routine practice.^9–11^ Continued investment in evidence-based approaches to training is needed to support doctors in conducting consultations using telehealth.^12,13^ Such support and training enables effective use of telehealth in practice, ensuring the doctor–patient relationship remains at the centre of care delivery. In detailing their multi-methods study design, White *et al* provide a glimpse into the future of telehealth research.^14^ This is achieved by bringing together different lenses to examine telehealth in practice, including using evidence to support co-design of guidelines.

The collection is completed with an editorial that considers the future of telehealth in Australia.^15^ Many of the considerations are transferable to other international health systems especially with regards to, for example, the need for ‘telehealth literacy’ among consumers and providers. Reflecting findings from articles in this collection, such an argument can be broadened to capture the entirety of the health system – that is, from the patient–provider consultation to the local and national governance. Improved telehealth literacy would be made possible through policies that not only fund increased access and infrastructure, but that also prioritise research and education.

Telehealth is a continuously evolving space, with technologies, priorities, policies, and doctor and patient preferences all changing at different paces. The articles in this collection capture a moment in time where health systems, still under pressure from an ongoing pandemic, explore the role and impact of telehealth as lessons from the necessary fast-paced adoption of telehealth become integrated into everyday care. As more research is conducted reflecting not only on what has occurred during the first years of the pandemic but also on the continued use of telehealth, there is an opportunity to critically appraise and refine how telehealth is managed at a system level to conduct consultations that enable increased access to services and allow for provision of high quality care.
